# Supplementary material for: Assessing the Clinical Effectiveness of Radioimmunotherapy with Combined Radionuclide/Monoclonal Antibody Conjugates in Cancer Treatment: Insights from Randomised Clinical Trials
Source: Cancers (Basel). 2025 Apr 23;17(9):1413. doi: 10.3390/cancers17091413 (PMC12071007; doi:10.3390/cancers17091413)
Supplement: Supplementary file 1 [file cancers-17-01413-s001.zip › cancers-3508603-supplementary.pdf]

Supplementary Files

Supplementary Table S1. Description of the database used and search terms

Databases	Search terms	Outcomes
PubMed	((radioimmunotherapy) OR (RIT)) AND ((cancer) OR (tumor) OR (patient) OR (treat*) OR (therap*) OR (tumour) OR (surviv*) OR (treatment outcome)) Filters: Full text, Clinical Trial, Randomized Controlled Trial, from 2000/1/1 - 2024/9	436 records
Scopus	TITLE-ABS-KEY ((radioimmunotherapy OR rit) AND (tumor OR cancer OR patient OR treat* OR therap* OR surviv* OR treatment AND outcome) AND (clinical AND trial)) AND PUBYEAR > 1999 AND PUBYEAR < 2025 AND PUBYEAR > 1999 AND PUBYEAR < 2025 AND (LIMIT-TO (DOCTYPE , "ar"))	427 records
EMBASE	1 ((radioimmunotherapy or RIT) and (tumor or cancer or surviv* or treat* or therap* or treatment outcome or patient) and clinical trial).mp. [mp=title, abstract, heading word, drug trade name, original title, device manufacturer, drug manufacturer, device trade name, keyword heading word, floating subheading word, candidate term word]<br>2 limit 1 to (full text and human and yr="2000 - 2024")	505 records
Cochrane Library (CENTRAL)	(radioimmunotherapy OR RIT):ti,ab,kw (Word variations have been searched) with Publication Year from 2000 to 2024, in Trials	412 records
Google scholar (first 24 pages)	"radioimmunotherapy" OR "RIT" "clinical trial" "patient" "cancer OR tumor" -model -animal -mice -review	461 records
Manual Search (PubMed, ResearchGate, Reference lists, ClinicalTrials.gov)	“radiolabled antibody”, “131I antibody”, “188Re antibody”, “zevalin”, “ibritumomab tiuxetan”, “90Y antibody”, “177lu antibody”, “licartin”, “bexxar”	102 records

Supplementary Table S2. Patient population treated with RIT in included clinical trials

**Clinical trial/study (year)**	**Study population**
Witzig et al (2002) [13]	$CD20^+$, R/R low-grade or transformed low-grade NHL patients.
Davis et al (2004) [14]	$CD20^+$, R/R low-grade or transformed low-grade NHL patients.
Verheijen et al (2006) [15]	Patients with EOC who had attained a complete clinical remission after cytoreductive surgery and platinum-based chemotherapy.
Wygoda et al (2006) [16]	Patients with high-grade gliomas after neurosurgery.
Xu et al (2007) [17]	Patients with advanced HCC after OLT.
Morschhauser et al (2008) [18]	$CD20^+$ FL patients who achieved a CR/CRu or PR after first-line induction treatment.
Goff et al (2009) [19]	$CD20^+$ FL (grade 1/2), patients who achieved a CR/CRu or PR after first-line induction treatment.
Sultana et al (2009) [20]	Patients with histologically/cytologically confirmed inoperable pancreatic head adenocarcinoma.
Shimoni et al (2012) [21]	$CD20^+$ aggressive NHL (including DLBCL or transformed FL), patients who failed to achieve CR with initial chemotherapy or relapsed after CR but were chemosensitive to second-line chemotherapy.
Press et al (2013) [22]	$CD20^+$, previously untreated, bidimensionally measurable bulky FL patients.
Vose et al (2013) [23]	Chemotherapy-sensitive persistent or relapsed DLBCL patients
Bian et al (2014) [24]	Barcelona Clinic Liver Cancer classification stage 0-B HCC patients.
Quackenbush et al (2015) [25]	Patients with FL who relapsed after chemotherapy.
Zhao et al (2016) [26]	Patients with stage II and IIIa NSCLC.
Li et al (2020) [27]	Patients who have underwent curative-intent resection of histologically confirmed HCC expressing CD147.
López-Guillermo et al (2022) [28]	Patients with FL who have achieved PR or CR/CRu after R-CHOP.
Tagawa et al (2023) [29]	Patients with high-risk non-metastatic castration-resistant prostate cancer.
Gyurkocza et al (2024) [30]	Elderly patients with R/R AML before alloHCT.
Ladetto et al (2024) [31]	Patients with R/R FL.
Laoruangroj et al (2024) [32]	Newly diagnosed, untreated asymptomatic low-tumor burden FL (grades 1 and 2) who did not require chemotherapy.

R/R: Relapsed or refractory; CR: Complete response; CRu: Unconfirmed complete response; PR: Partial response; CVP/COP: Cyclophosphamide, vincristine, and prednisone; CHOP: Cyclophosphamide, doxorubicin, vincristine, and prednisone; R-CHOP: Rituximab, cyclophosphamide, doxorubicin, vincristine, and prednisone; alloHCT: Allogeneic hematopoietic cell transplantation; OLT: Orthotopic liver transplantation; PET: Positron Emission Tomography.

Supplementary Table S3. Quality score assessment for included clinical trials

	Q1	Q2	Q3	Q4	Q5	Q6	Q7	Q8	Q9	Q10	Q11	Q12	Q13	Overall score
Witzig et al [13]	R	R	✓	R	R	✓	✓	✓	✓	✓	✓	✓	✓	11
Davis et al [14]	R	R	✓	R	✕	✓	✓	✓	✓	✓	✓	✓	✓	10.5
Verheijen et al [15]	R	R	✓	✕	✕	✓	R	✓	R	✓	✓	✓	✓	9
Wygoda et al [16]	R	R	✓	R	R	R	R	R	R	✓	R	R	✓	8
Xu et al [17]	✓	✓	✓	✓	✓	✓	✓	✓	R	✓	✓	✓	✓	12.5
Morschhauser et al [18]	R	R	✓	✕	✕	✓	✓	✓	R	✓	✓	✓	✓	9.5
Goff et al [19]	✓	R	✓	R	R	✓	R	✓	R	✓	✓	✓	✓	10.5
Sultana et al [20]	✓	R	R	✕	✕	✓	R	✓	R	✓	✓	✓	R	8.5
Shimoni et al [21]	R	R	✓	✕	✕	✓	R	✓	R	✓	R	✓	✓	8.5
Press et al [22]	✓	✕	✓	✕	✕	✓	✓	✓	✓	✓	✓	✓	✓	10
Vose et al [23]	R	R	✓	✕	✕	✓	R	✓	R	✓	✓	✓	✓	9
Bian et al [24]	✓	R	✓	R	R	✓	✓	✓	R	✓	✓	✓	✓	11
Quackenbush et al [25]	R	R	✓	✕	✕	✓	R	✓	R	✓	✓	✓	✓	9
Zhao et al [26]	R	R	✓	R	R	✓	R	✓	R	✓	✓	✓	✓	10
Li et al [27]	✓	✓	✓	✕	✕	✓	✓	✓	✓	✓	✓	✓	✓	11
López-Guillermo et al [28]	R	R	✓	✕	✕	✓	R	✓	R	✓	✓	✓	✓	9
Tagawa et al [29]	R	R	✓	✓	✓	✓	R	✓	R	✓	✓	✓	✓	11
Gyurkocza et al [30]	R	R	✓	✕	✕	✓	R	✓	R	✓	✓	✓	✓	9
Ladetto et al [31]	✓	R	✓	✕	✕	✓	R	✓	R	✓	✓	✓	✓	9.5
Laoruangroj et al [32]	R	R	✓	✕	✕	✓	R	✓	R	✓	✓	✓	✓	9

✓ = yes (= 1), ✕ = no (= 0), R = unclear (= 0.5) ; quality assessment decision rules for 13 scales [(i) overall score ⩽6: poor, (ii) overall scores = 7 to 10: medium, and (iii) overall score>10: high]
